# Efficiency of reverse genetics methods for rescuing severe acute respiratory syndrome coronavirus 2

**DOI:** 10.71150/jm.2411023

**Published:** 2025-02-27

**Authors:** Chang-Joo Park, Taehun Kim, Seung-Min Yoo, Myung-Shin Lee, Nam-Hyuk Cho, Changhoon Park

**Affiliations:** 1 Department of Microbiology and Immunology, Eulji University School of Medicine, Daejeon 35233, Republic of Korea; 2 Department of Microbiology and Immunology, Seoul National University College of Medicine, Seoul 03083, Republic of Korea

**Keywords:** circular polymerase extension reaction, infectious clone, infectious sub-genomic amplicons, reverse genetics, SARS-CoV-2

## Abstract

Bacteria-free reverse genetics techniques are crucial for the efficient generation of recombinant viruses, bypassing the need for labor-intensive bacterial cloning. These methods are particularly relevant for studying the pathogenesis of severe acute respiratory syndrome coronavirus 2 (SARS-CoV-2), the causative agent of COVID-19. This study compared the efficiency of three bacteria-free approaches—circular polymerase extension reaction (CPER) with and without nick sealing and infectious sub-genomic amplicons (ISA)—to bacterial artificial chromosome (BAC)-based technology for rescuing SARS-CoV-2. Significant differences in viral titers following transfection were observed between methods. CPER with nick sealing generated virus titers comparable to those of the BAC-based method and 10 times higher than those of the standard CPER. In contrast, ISA demonstrated extremely low efficiency, as cytopathic effects were detected only after two passages. All rescued viruses exhibited replication kinetics consistent with those of the original strain, with no significant deviation in replication capacity. Furthermore, the utility of CPER and ISA in genetically modifying SARS-CoV-2 was demonstrated by successfully inserting the gene encoding green fluorescent protein into the genome. Overall, this study underscores the potential of bacteria-free methods, such as CPER and ISA, in advancing SARS-CoV-2 research while highlighting their significant differences in efficiency.

## Introduction

SARS-CoV-2, which emerged in Wuhan, China (Wu et al., 2020), has caused over 775 million cases and more than 7 million deaths worldwide (WHO, 2024). The virus shares similarities with SARS and MERS but is more transmissible (Zhou et al., 2021). It possesses a typical coronavirus genome structure, comprising a 29.9 kb single-stranded RNA encoding four major structural proteins and several accessory proteins essential for viral replication, immune evasion, and assembly (Redondo et al., 2021; Zhou et al., 2021). However, pathogenesis remains unclear.

Reverse genetics technology is critical for studying viral pathogenesis and developing vaccines (Hou et al., 2020). However, traditional reverse genetic techniques for constructing a full-length infectious cDNA clone of coronaviruses have previously reported severe mutations in the viral sequence, such as unanticipated insertions or deletions when propagated as cloned cDNA in bacteria, due to the large genomic size and sequence instability (Almazan et al., 2014). Alternatively, Bacterial artificial chromosome (BAC) was first used to generate a full-length infectious clone of the coronavirus transmissible gastroenteritis virus (TGEV) (Almazán et al., 2000; González et al., 2002). This approach uses a low-copy-number BAC plasmid to minimize toxicity caused by unstable coronavirus sequences in bacteria (Wang et al., 1997). BAC have also been used to rescue recombinant SARS-CoV-2 (Chiem et al., 2021; Ye et al., 2020).

Bacteria-free methods have been developed to address these challenges. Even the BAC system requires significant time and effort to construct an infectious clone including whole genome of coronavirus, as the creation of the clone inevitably involves plasmid amplification in bacterial systems. In contrast, novel PCR-based techniques offer viable alternatives by completely eliminating the need for bacterial amplification. By bypassing this labor-intensive process, the rapid rescue of designed viruses becomes feasible. Among bacteria-free approaches, infectious sub-genomic amplicon (ISA) and circular polymerase extension reaction (CPER) are widely regarded as the most promising techniques.

The ISA method uses PCR to create overlapping viral genome fragments, allowing for natural recombination within cells (Aubry et al., 2014; Park et al., 2023). An alternative technique, CPER, refines the PCR to amplify fragments that encompass the viral genome. By ensuring that the first and last fragments have overlapping ends, this method promotes their annealing, followed by extension through DNA polymerase using adjacent fragments as primers. This series of reactions yields a circular infectious clone of the full viral genome, prepared for direct transfection into susceptible cells for virus rescue (Edmonds et al., 2021). It accomplishes the full genomic cDNA ligation during the reaction, unlike the ISA, which completes the genome assembly during transcription within the cells. Both methods have been successfully used to rescue SARS-CoV-2, although their efficiencies remain to be evaluated (Amarilla et al., 2021; Mélade et al., 2022; Torii et al., 2021).

In this study, we compared ISA and CPER for rescuing recombinant SARS-CoV-2 based on post-transfection viral titers. CPER demonstrated superior efficiency compared to ISA, as confirmed by cytopathic effects (CPE) after two passages. Furthermore, CPER could be improved by adding a complementary step of 5′ phosphorylation and ligation to seal nicks on the circular infectious clone. The modified CPER method exhibited efficiency comparable to that achieved with cDNA produced by BAC cloning. These findings provide insights into the relative efficiencies of these two bacteria-free methods, offering guidance for researchers in selecting the most effective approach for reverse genetics of coronaviruses in future pandemics. 

## Materials and Methods

### Virus and cells

SARS-CoV-2 strain (NCCP No. 43326) was provided by the National Culture Collection for Pathogens (NCCP). The virus was isolated by the Korea Disease Control and Prevention Agency in 2020 (Chung et al., 2022). The whole genome sequence has been deposited at GenBank (No. MW466791). Comparative genome analysis revealed that the virus has four nucleotide and two amino acid mutations compared to the protype SARS-CoV-2 strain, Wuhan-Hu-1 (No. NC045512), with difference at 781 aa of NSP3 (Leu > Phe) and at 84 aa of ORF8 (Leu > Ser). The virus was used for RNA extraction, cDNA synthesis, PCR amplification, and comparing replication kinetics.

Baby-hamster kidney 21 (BHK-21, Korea Cell Line Bank No. 10010) cells were used for ISA transfection and viral rescue. Vero E6 cells (ATCC CRL-1586) were cultured for BAC, CPER, CPER-NS infectious clone transfection, virus rescue, passaging, and titration. The cells were maintained in Dulbecco's Modified Eagle Medium (Thermo Fisher Scientific) supplemented with 10% fetal bovine serum (FBS) and 1% penicillin-streptomycin (P/S), as previously described. Experiments for RNA extraction, transfection, virus rescue, passaging, and titration were performed in a BSL3 laboratory of Seoul National University.

### Reverse genetics design and virus rescue

The SARS-CoV-2 (NCCP No. 43326) genome was divided into seven fragments prepared via de novo synthesis or PCR. The first fragment included the cytomegalovirus (CMV) promoter and hammerhead ribozyme sequence placed before the 5′ UTR of the virus, while the last fragment contained the hepatitis delta virus (HDV) ribozyme sequence added after the 3′ UTR of the viral genome. Each fragment was designed with 60–80 bp overlapping regions.

**Fig. 1.**
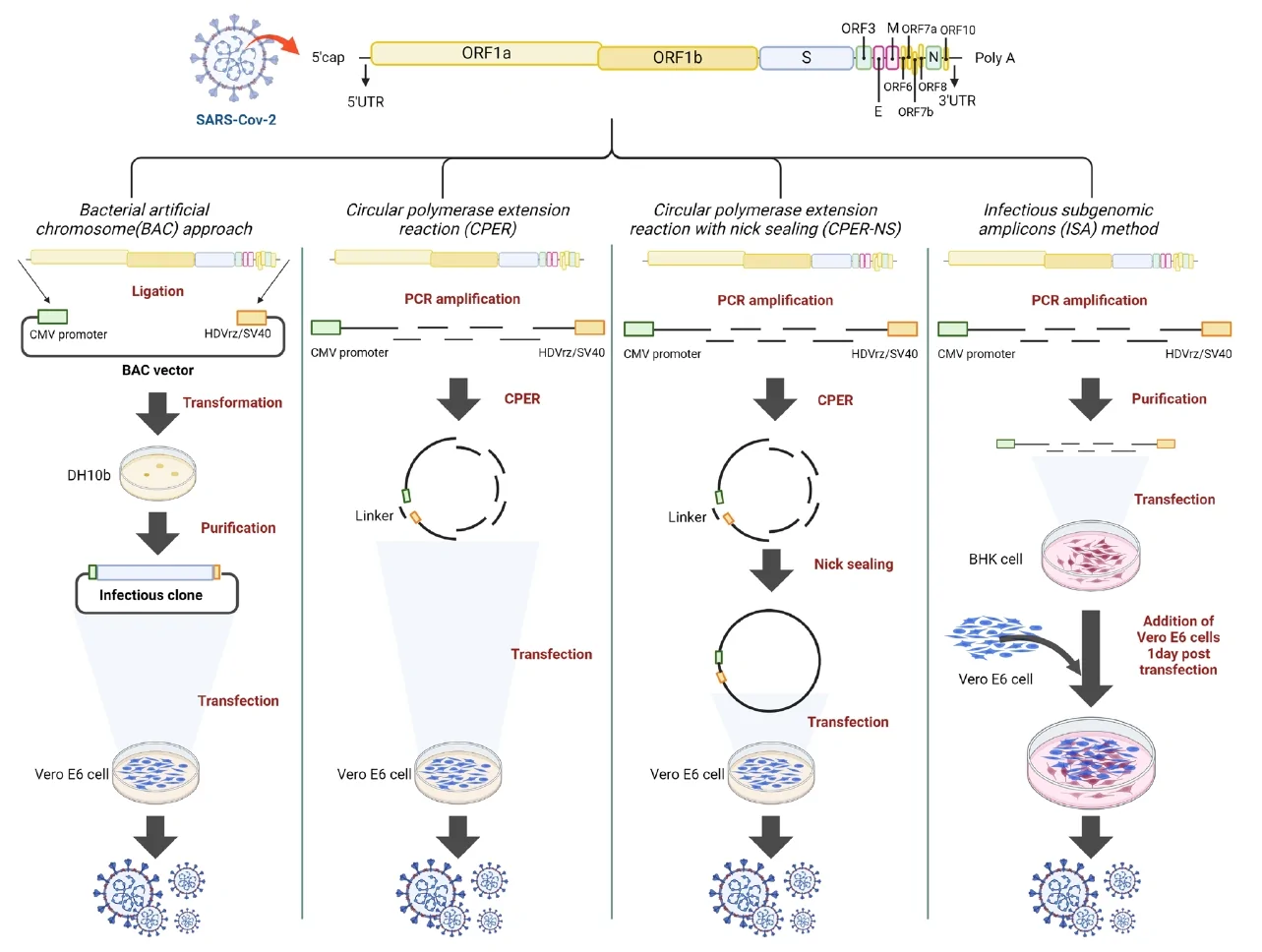
Schematic diagram of reverse genetics methods used in this study. BAC method: An infectious clone was constructed by ligating fragments covering the entire viral genome. After amplification by *E. coli* propagation, the clone was transfected into Vero E6 cells. CPER method: Fragments were prepared by PCR amplification and CPER was used to assemble a circular clone, which was then transfected into Vero E6 cells. CPER-NS method: An additional step was applied to eliminate nicks in the circular clones produced by CPER. ISA method: Fragments covering the entire viral genome were directly transfected into BHK cells, followed by the addition of Vero E6 cells.

Four reverse genetic methods were employed to rescue the virus (Fig. 1). The first method, the BAC approach, involves inserting the entire viral genome into a BAC plasmid, amplifying the infectious clone in DH10b *E. coli*, and purifying it. The second method, ISA, involves amplifying the seven fragments covering the viral genome by PCR and purifying them. The third method, CPER, utilized six fragments same with those in the ISA method. However, the last fragment had overlapping ends (52 bp) with the first fragment, achieved using a specifically designed reverse primer (CPER-R). Additional modified PCRs facilitate the annealing and extension by DNA polymerase, resulting in a circular infectious clone of the full viral genome. The fourth method, CPER with nick sealing (CPER-NS), followed the same CPER procedure but included an additional step with T4 kinase and Taq polymerase to eliminate nicks. The prepared constructs were then transfected into Vero E6 or BHK cells using the same transfection reagent and protocol. The differences in purity and concentration among the constructs were confirmed (Table 1).

### Fragment preparation

The first and last fragments were synthesized de novo based on the confirmed sequence and amplified using specific primers (Table 1). For the last fragment, the CPER and CPER-NS employed an extended reverse primer designed to ligate the first fragment, producing a circular infectious clone after CPER. Aside from the last fragment, all fragments were amplified using the same primer pairs across CPER, CPER-NS, and ISA.

For preparing fragments 2, 3, 4, 5, and 6, viral RNA was extracted from the cell supernatants using the Viral Gene-Spin™ kit (Intron Biotechnology). Subsequently, cDNA was synthesized using the PrimeScript™ RT Reagent Kit (Takara Bio), amplified using PrimeSTAR GXL DNA Polymerase (TaKaRa Bio), and purified using the MiniBEST DNA Fragment Purification Kit Ver.4.0 (TaKaRa Bio).

To construct a strain expressing a fluorescent protein (EGFP), the last fragment sequence was modified. Specifically, the EGFP sequence replaced the region corresponding to ORF7a, starting from the ATG codon up to 720 nucleotides. The reporter gene was positioned immediately before the end of ORF6, preceded by a TRS and followed by a TAA stop codon. This modification was achieved through a cloning procedure, involving digestion with the PpuMI and PsiI restriction enzymes (NEB) and subsequent ligation using T4 ligase (NEB). The modified last fragment containing the EGFP sequence was then employed in CPER, CPER-NS, and ISA to rescue the fluorescent SARS-CoV-2.

### CPER and nick sealing

The prepared DNA fragments, each at a molar concentration of 0.1 pmol, were combined with PrimeSTAR GXL DNA Polymerase (TaKaRa Bio) and subjected to PCR cycling conditions as previously described (Torii et al., 2021). Briefly, the reactions started with an initial denaturation at 98°C for 2 min, followed by 20 cycles of 98°C for 30 s, 55°C for 30 s, and 68°C for 30 min, with a final extension at 68°C for 30 min. The reaction mixture was then directly used for transfection.

To optimize CPER efficiency, an additional nick sealing step was introduced as previously described (Liu & Gack, 2023). Each fragment was 5′-phosphorylated in 50 μl reaction mixture containing T4 polynucleotide kinase (NEB) and 10 mM ATP, incubated for 30 min at 35°C and 20 min at 65°C. After purification, the CPER was performed under the same conditions as described above. The product was then subjected to a nick sealing process, which involved incubation at 50°C for 1 h and 60°C for 1 h in 50 μl reaction mixture containing 1 mM β-nicotinamide adenine dinucleotide (NAD+) (NEB) and HiFi Taq DNA ligase (NEB). The final product was transfected into Vero E6 cells, which had been seeded into 6-well plates containing 70% confluent cells.

### Cell transfection

The BAC encoding the entire SARS-CoV-2 genome was purchased from VectorBuilder Inc. (Catalog No: Ecoli: pBACYAC-SARS-Cov-2 [NC_045512.2]). After transforming DH10b cells and amplifying the bacteria, the plasmid was isolated using the NucleoBond Xtra Midi EF kit (Macherey-Nagel) to obtain transfection-grade DNA.

The transfection was performed following previously reported protocols (Chiem et al., 2021; Liu & Gack, 2023; Mélade et al., 2022; Torii et al., 2021; Ye et al., 2020). Briefly, 2 µg of the BAC-infectious clone, 25 µl of CPER product, 25 µl of CPER-NS product, and 4 µg of ISA fragments were each diluted in Opti-MEM medium (Life Technologies). Separately, 12.5 µl of Lipofectamine LTX (Life Technologies) was diluted in an equal volume of Opti-MEM medium. These solutions were then combined and incubated at 25°C for 1 h. Subsequently, the mixtures containing BAC-infectious clone, CPER product, and CPER-NS product were added to a six-well plate containing Vero E6 cells at 70% confluency and incubated for 5 h. Meanwhile, the ISA fragment–lipid complex was added to a 50% confluent BHK cell culture plate and incubated for 15 h before replacing the medium and adding 1×10^5^ Vero E6 cells. After replacing the supernatant with fresh medium, the cells were incubated for 6 d at 37°C with 5% CO_2_. The cell supernatant (passage 0) was then serially passaged. The passages were performed by inoculating 1 ml cell supernatant, purified by centrifugation and 0.45-µm filtration, into a T25 flask containing 70% confluent Vero E6 cells.

### Virus titration

After transfection, 100 µl supernatant was collected to quantify the rescued SARS-CoV-2 using a foci-forming assay (FFA) with Vero E6 cells. The cells were seeded in a 24-well plate and incubated overnight at 37°C with 5% CO_2_. When the cells reached 90% confluency, they were subjected to serum starvation for 1 h. During this time, the supernatant was serially diluted 10-fold with serum-free Dulbecco’s Modified Eagle Medium (DMEM; Welgene).

After the starvation period, the medium was replaced with the diluted supernatants and the cells were incubated for 1.5 h. The cells were then washed and overlaid with DMEM containing 1% methylcellulose (Sigma), 2% FBS (Gibco), and P/S (Gibco). After plaque formation, the cells were fixed with methanol (Merck) and 4% paraformaldehyde (PFA; CellNest), then immunostained overnight with an anti-SARS-CoV-2 nucleocapsid antibody (Abfrontier). A secondary alkaline phosphatase-conjugated antibody (Invitrogen) was added for 1 h, followed by a 10-min incubation with NBT/BCIP substrate (Thermo Scientific). The stained foci were counted after rinsing the cells with PBS.

### Virus replication kinetics

For comparison with the original viral growth, three-passaged recombinant SARS-CoV-2 viruses were inoculated into Vero E6 cells at 0.1 multiplicity of infection (MOI) of 1.5 h. After incubation, cells were washed and cultured in DMEM containing 2% FBS and penicillin/streptomycin. Supernatants were collected at 0, 12, 24, 36, and 48 h post-infection to determine the viral titers. FFA was performed as described above.

### Statistical analysis

Statistical analyses were performed using GraphPad Prism version 8.4.3. Viral titers were compared using either Student’s *t*-test or one-way ANOVA followed by Tukey's post-hoc adjustment. The results are expressed as the mean ± standard deviation for each group. Statistical significance was set at p < 0.05.

## Results and Discussion

### The virus rescue efficiency differed significantly between the reverse genetics methods

**Fig. 2.**
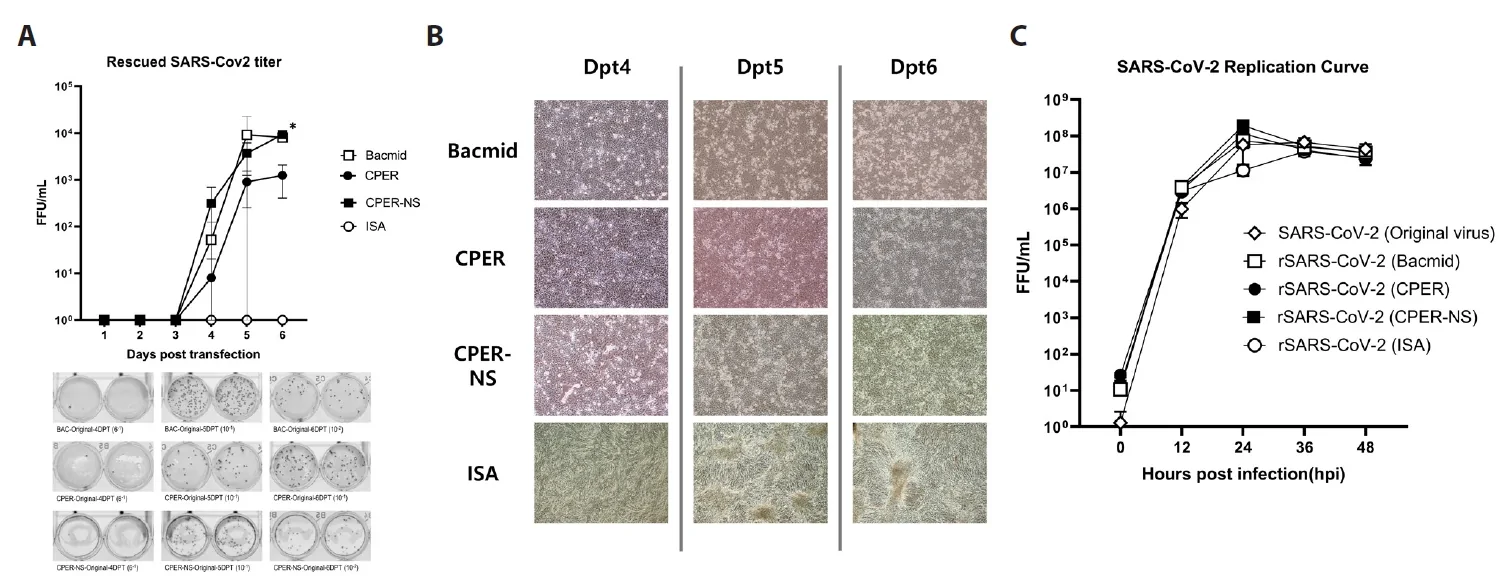
Efficiency of the tested methods. (A) Virus titer after transfection: The BAC and CPER-NS methods showed significantly higher viral titers than the CPER and ISA methods at 6 dpt (* indicates significant difference with a *P* value < 0.05). The ISA method did not result in viral rescue until six dpt. (B) Cytopathic effect (CPE) by the rescued virus: CPE was observed 4 days post-transfection in wells treated with BAC, CPER-NS, and CPER. (C) Replication curve of passaged viruses: No significant difference was observed between the replication abilities of the rescued and original viruses.

The efficiency of each method was determined by quantifying the viruses rescued from the supernatant after transfection. Using FFU assays with Vero E6 cells, the virus was detected in the supernatant from day 4 post-transfection using BAC, CPER, and CPER-NS. Virus rescue efficiency with the BAC approach increased from 52 FFU/ml on day 4 to 8,067 FFU/ml by day 6. For CPER, the rescue efficiency rose from 8 FFU/ml on day 4 to 1,247 FFU/ml on day 6, while for CPER-NS, it increased from 316 FFU/ml on day 4 to 9,267 FFU/ml on day 6. The last titer of the virus rescued by day 6 was significantly higher in CPER-NS and BAC, on average 10 times higher than that in CPER. However, ISA did not yield any FFU counts over the 6-day period (Fig. 2A). CPEs were observed from day 4 post-transfection using all methods except ISA. No CPE was confirmed in cells treated with ISA over the 6-day period, indicating lower efficiency than other methods (Fig. 2B). After two passages, CPE was observed in Vero E6 cells infected with the rescued virus using ISA.

### The replication ability and sequence of the rescued SARS-CoV-2 strains were consistent with those of the original strain

The replication ability of the rescued viruses was compared with that of the original strain. After transfection, the three passaged strains were inoculated into Vero E6 cells at 0.1 MOI, and the FFU was calculated every 12 h. For the original strain, the average FFU/ml reached 116,000 at 12 h, 16.6 million at 24 h, 39 million at 36 h, and 14.8 million at 48 h. The strain rescued using the BAC method showed an average FFU/ml of 574,000 at 12 h, 44 million at 24 h, 36 million at 36 h, and 54 million at 48 h. The CPER-rescued strain showed values of 384,000 at 12 h, 29.6 million at 24 h, 22.6 million at 36 h, and 14.2 million at 48 h. The strain rescued using the CPER-NS method recorded 558,000, 32.2 million, 44.4 million, and 28 million FFU/ml at 12, 24, 36, and 48 h, respectively. The ISA-rescued strain reached 432,000 at 12 h, 40.2 million at 24 h, 37.6 million at 36 h, and 20.6 million at 48 h. Viruses rescued using the four methods showed no significant differences from the original strain at every time point (Fig. 2C).

To determine the sequence differences between the rescued viruses, the full-length genome of the second passage strains was sequenced. The results revealed only one mutation in the passaged viral genome. Specifically, the strains obtained using CPER and ISA exhibited a mutation from C to T at nucleotide position 9195, resulting in a threonine to isoleucine change at position 214 of NSP4. Despite this mutation, the replication curves of the mutated strains did not differ significantly from that of the original strain, indicating that the mutation is unlikely to affect viral propagation.

### Fluorescent SARS-CoV-2 was successfully generated using the reverse genetics methods

**Fig. 3.**
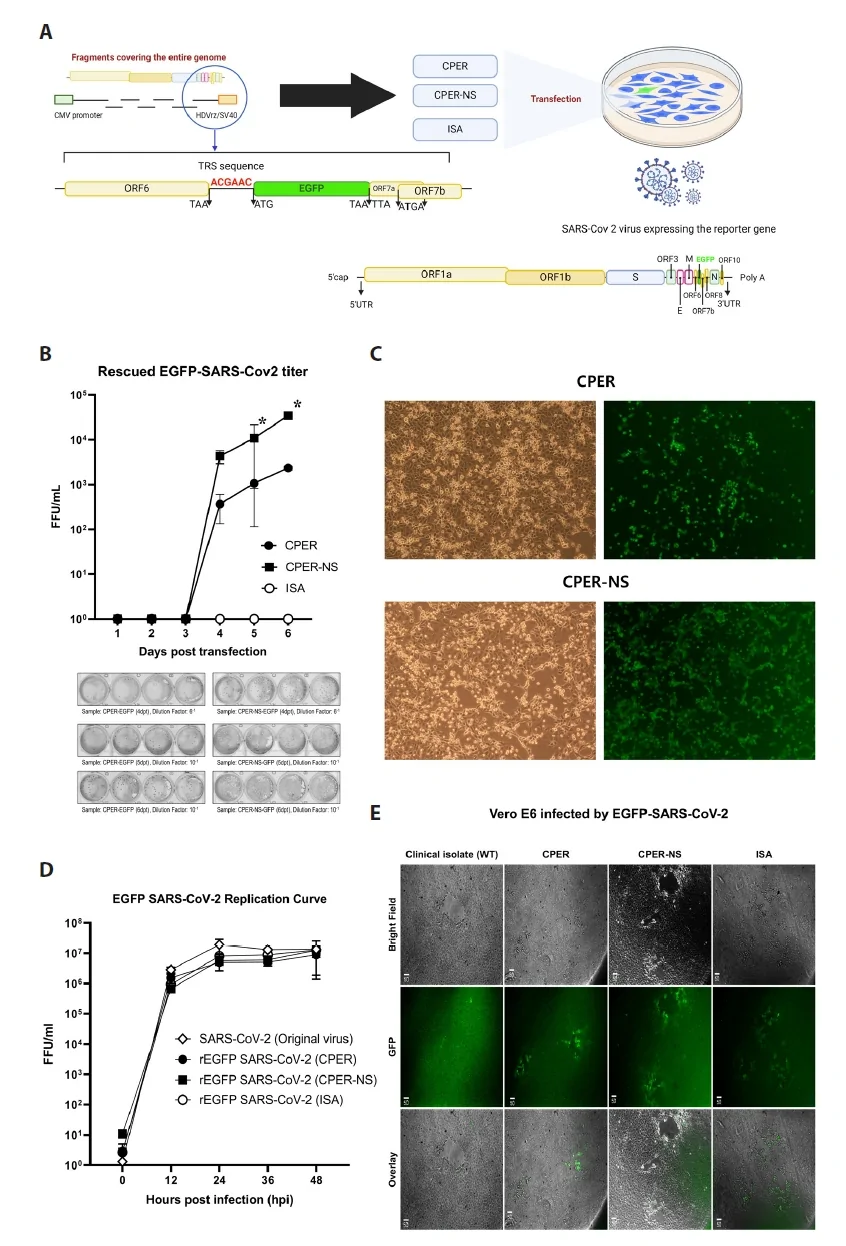
Production and replication ability of foreign gene-expressing SARS-CoV-2. (A) Reverse genetics design for EGFP-expressing SARS-CoV-2: The foreign gene (EGFP) was inserted into the ORF7a region of the viral genome. This modified fragment was used in three reverse genetic methods: CPER, CPER-NS, and ISA. (B) Virus titer after transfection: CPER-NS showed significantly higher EGFP-SARS-CoV-2 titers than the CPER and ISA methods at 5 and 6 dpt (* indicates a significant difference with a *P* value < 0.05). The ISA method did not result in viral rescue until 6 dpt. (C) CPE and fluorescence confirmation: CPE and fluorescence were observed in wells treated with CPER-NS and CPER at 6 dpt. (D) Replication curve of the passaged viruses. There was no significant difference in the replication ability between the passaged fluorescent viruses and the original virus. (E) FFU assay: Focus-forming units (FFU) were observed in wells inoculated with passaged fluorescent viruses.

CPER, CPER-NS, and ISA were used to produce fluorescent SARS-CoV-2 by inserting the EGFP sequence into ORF7a (Fig. 3A). The efficiency of each method was determined by quantifying the virus rescued from the supernatant after transfection (Fig. 3B). Green fluorescence was detected in the transfected cells from day 4 post-transfection using CPER and CPER-NS (Fig. 3C). Using an FFU assay with Vero E6 cells, the fluorescent virus rescued by the CPER method increased in average FFU/ml from 366 on day 4 to 2,340 on day 6, while the virus rescued by the CPER-NS method increased from 4,320 on day 4 to 34,500 on day 6. The FFU assay showed that the CPER-NS method was significantly more efficient in rescuing the recombinant virus compared to the CPER method on days 5 and 6 post-transfection. (Fig. 3B). However, ISA did not show any FFU counts over the 6-day period. After two passages, fluorescence was detected in Vero E6 cells infected with the recombinant virus generated using the ISA method.

The replication ability of the reporter gene-expressing viruses was compared with that of the original strain. After transfection, the three passaged strains were inoculated into Vero E6 cells at 0.1 MOI and the FFU was calculated every 12 h. For the original strain, the average FFU/ml at 12, 24, 36, and 48 h was 2.83 million, 19.2 million, 12.93 million, and 13.46 million, respectively. The strain rescued using the CPER method showed average FFU/ml values of 1.52 million at 12 h, 4.93 million at 24 h, 5.06 million at 36 h, and 8.8 million at 48 h. The strain rescued using the CPER-NS method recorded 666,000, 5.73 million, 6 million, and 12.53 million FFU/ml at 12, 24, 36, and 48 h, respectively. The strain rescued using the ISA method reached 933,000, 8 million, 8.8 million, and 12.8 million FFU/ml at 12, 24, 36, and 48 h, respectively. The viruses rescued using the four methods showed no significant differences from the original strain at every time point (Fig. 3D & 3E).

Reverse genetics has proven to be an indispensable tool in virology and its utility has been demonstrated across a diverse array of viral pathogens (Almazán et al., 2014; Masters, 1999). However, the large size and inherent instability of the SARS-CoV-2 genome pose significant challenges to the rapid application of reverse genetics using traditional methods. Despite these obstacles, recent studies have reported successful viral generation using various methodologies including BAC, CPER, and ISA (Amarilla et al., 2021; Chiem et al., 2021; Mélade et al., 2022; Torii et al., 2021; Ye et al., 2020).

In this study, the four reverse genetics methods employed utilized a common expression system, which incorporated a sequence format consisting of a CMV promoter, a hammerhead ribozyme sequence upstream of the viral genome’s 5′ UTR, and an HDV ribozyme sequence downstream of the 3′ UTR (Almazán et al., 2014). Therefore, the efficiency of viral rescue by these methods may depend on the presence of a fully assembled genome. Among these methods, BAC exhibited the highest virus rescue efficiency, owing to the transfection of an already completed infectious clone. However, the labor-intensive and time-consuming nature of the cloning process remains a significant drawback (Chiem et al., 2021; Ye et al., 2020).

However, CPER and ISA rely on PCR amplification, offering a simpler and faster alternative to BAC by eliminating the need for genome amplification in *E. coli* (Amarilla et al., 2021; Mélade et al., 2022; Torii et al., 2021). In this study, full sequencing of the rescued viruses confirmed that CPER and ISA not only expedited the process but also effectively mitigated mutation-related issues, a known limitation of bacteria-free reverse genetics techniques (Cai & Haung, 2023). The use of a high-fidelity PCR polymerase in these methods minimized mutations, and the replication curves of the mutated strains did not differ significantly from those of the original strain, suggesting that the introduced mutations are unlikely to affect viral propagation. Therefore, CPER and ISA are viable options for reverse genetics, particularly during pandemics.

Nevertheless, our comparative evaluation revealed significant differences in rescue efficiency between CPER and ISA. A key technical distinction is that CPER achieves complete viral genome ligation during the reaction, whereas ISA relies on in cellular recombination for genome assembly (Edmonds et al., 2013). The observed discrepancies may be attributed to the lower efficiency of recombination of the seven genome fragments in ISA compared to the in vitro transfection of a pre-assembled genome clone. Our previous work on dengue viruses showed comparable efficiencies of ISA and CPER in rescuing viruses (Park et al., 2023; Tamura et al., 2022). These contrasting findings suggest that ISA efficiency is particularly sensitive to the size and number of fragments constituting the viral genome, as the Flaviviridae genome (~11 kb) is almost three times smaller than the SARS-CoV-2 genome (~30 kb). Although ISA is a convenient method, its efficiency diminishes significantly when larger genomes and more fragments are involved, unlike methods, such as BAC or CPER, which involve genome pre-assembly in vitro.

The ISA method employed a distinct two-step transfection process in this study, which involved initial transfection in BHK cells, followed by inoculation with Vero E6 cells. This two-step approach is critical for successfully rescuing SARS-CoV-2. Our previous attempts to independently transfect BHK or Vero E6 cells failed to yield rescued virus (data not shown), highlighting the necessity of combining these cell types. Typically, BHK cells are characterized by high transfection efficiency but have limited capacity for SARS-CoV-2 replication and propagation. Conversely, Vero E6 cells exhibit lower transfection efficiency but support robust viral replication and propagation (Dighe et al., 2022). Consequently, a study utilizing the ISA method for SARS-CoV-2 adopted this two-step strategy (Mélade et al., 2022).

Consistent with previous studies, our results indicate that CPER and ISA are generally less efficient than BAC (Amarilla et al., 2021; Mélade et al., 2022). However, our findings also suggest that with appropriate optimizations such as those introduced in CPER-NS, rescue efficiencies comparable to BAC can be achieved. A prior study on CPER-NS demonstrated that virus rescue could be achieved 2 days faster (as early as 3 days post-transfection) compared to standard CPER, while maintaining comparable viral yields (5×10⁵ – 10⁶ PFU/ml) (Liu & Gack, 2023). Extending the understanding of CPER limitations, our study focused on addressing its intrinsic challenge, the presence of staggered nicks that hinder efficient expression in mammalian cells. By quantifying the rescued viruses over a timeline post-transfection, we observed that CPER-NS achieved nearly 10-fold higher rescue efficiency compared to standard CPER.

In this study, we evaluated the efficiency of various reverse genetics techniques for SARS-CoV-2 detection by comparing the post-transfection viral titers. Our findings provide a useful framework for selecting appropriate methods for future coronavirus research. Given the persistent threat posed by emerging viral pathogens, the ability to rapidly generate recombinant viruses is essential. These results highlight the need for ongoing optimization of reverse genetics methodologies to ensure preparedness for future pandemics and advance virological research and public health responses.

## Figures and Tables

**Table 1.** PCR primers to produce fragments and clones in transfection mixture

Fragment	Primers			Size
1	*F : atagtaatcaattacggggtcattag*			7,900 bp
	*R : ttgcagccaatccaagtaca*			
2	*F : tttggcttagttgcagagtgg*			2,754 bp
	*R : gagagcctttgcgagatgac*			
3	*F : tggagcaatggatacaactagc*			3,340 bp
	*R : acggcagtacagacaacacg*			
4	*F : cagttacaccggaagccaat*			3,108 bp
	*R : cgtatgcaagcaccacatct*			
5	*F : ctgttggggcttgtgttctt*			5,130 bp
	*R : ttggatttgtattcctccaaaa*			
6	*F : caaaccacgcgaacaaatag*			4,132 bp
	*R :agttccaattgtgaagattctcataaacaaatccataagttcgtttatgtgtaatgtaatttgactcctttgagcactggctcagagtcgtcttcatcaaatttgcagcag*			
	*gatccacaagaacaacagc*			
7	*F : ctctgagccagtgctcaaagga*			4,957 bp
	*R : cgtcgacgagaataactataacggtcctaaggt*			
	*CPER-R : gcggaactccatatatgggctatgaactaatgaccccgtaattgattactatagaataactataacggtcctaaggt*			5,009 bp
**Virus rescue after transfection (passage 0)**				
**Reverse genetics**		**Transfected DNA**	**Transfected cell line**	**CPE**
Bacmid approach		2 µg	Vero E6	Positive
CPER		25 µl mixture of CPER reaction	Vero E6	Positive
CPER-NS		25 µl mixture of CPER & Nick sealing reaction	Vero E6	Positive
ISA		Total 4ug (Fragments molar ratio = 1:1:1:1:1:1:1)	BHK21 +Vero E6	Negative
